# Supplementary material for: From sequence to enzyme mechanism using multi-label machine learning
Source: BMC Bioinformatics. 2014 May 19;15:150. doi: 10.1186/1471-2105-15-150 (PMC4229970; doi:10.1186/1471-2105-15-150)
Supplement: Additional file 2 — Java code of ml2db. Additional file ml2db_code.tar.gz contains the Java source code to run the multi-label machine learning experiments and save the results to database. The code’s Javadoc is included. [file 1471-2105-15-150-S2.zip › additional file 2/ml2db/ml2db/doc/test/ml2db/al/utility/neighbourhood/LinearNNSearchSkipInstanceItselfTest.html]

LinearNNSearchSkipInstanceItselfTest


JavaScript is disabled on your browser.


- Overview
- Package
- Class
- Use
- Tree
- Deprecated
- Index
- Help

- Prev Class
- Next Class

- Frames
- No Frames

- All Classes

- Summary:
- Nested |
- Field |
- Constr |
- Method

- Detail:
- Field |
- Constr |
- Method


test.ml2db.al.utility.neighbourhood

## Class LinearNNSearchSkipInstanceItselfTest

- java.lang.Object
- - junit.framework.Assert
  - - junit.framework.TestCase
    - - test.ml2db.al.utility.neighbourhood.LinearNNSearchSkipInstanceItselfTest

- All Implemented Interfaces:
  :   junit.framework.Test

  ---

    

  ```
  public class LinearNNSearchSkipInstanceItselfTest
  extends junit.framework.TestCase
  ```

  Class

  Version:
  :   5 Oct 2012

  Author:
  :   Luna De Ferrari ldf(at)st-andrews.ac.uk or
      luna.deferrari@st-andrews.ac.uk

- - ### Constructor Summary

    Constructors

    Constructor and Description

    `LinearNNSearchSkipInstanceItselfTest()`
  - ### Method Summary

    Methods

    | Modifier and Type | Method and Description |
    | --- | --- |
    | `void` | `testGetNeighbours()` Test method for `main.ml2db.al.utility.neighbours.LinearNNSearchSkipInstanceItself#getNeighbours(weka.core.Instance, uk.ac.ed.inf.enzml.mulan.mulanarff.IdMultiLabelInstances, int, boolean)` . |
    | `void` | `testKNearestNeighboursInstanceIntBoolean()` Test method for `LinearNNSearchSkipInstanceItself.kNearestNeighbours(weka.core.Instance, int, boolean)` . |
    | `void` | `testLinearNNSearchSkipInstanceItself()` Test method for `main.ml2db.al.utility.neighbours.LinearNNSearchSkipInstanceItself#LinearNNSearchSkipInstanceItself(uk.ac.ed.inf.enzml.mulan.mulanarff.IdMultiLabelInstances)` . |

    - ### Methods inherited from class junit.framework.TestCase

      `countTestCases, getName, run, run, runBare, setName, toString`
    - ### Methods inherited from class junit.framework.Assert

      `assertEquals, assertEquals, assertEquals, assertEquals, assertEquals, assertEquals, assertEquals, assertEquals, assertEquals, assertEquals, assertEquals, assertEquals, assertEquals, assertEquals, assertEquals, assertEquals, assertEquals, assertEquals, assertEquals, assertEquals, assertFalse, assertFalse, assertNotNull, assertNotNull, assertNotSame, assertNotSame, assertNull, assertNull, assertSame, assertSame, assertTrue, assertTrue, fail, fail, failNotEquals, failNotSame, failSame, format`
    - ### Methods inherited from class java.lang.Object

      `equals, getClass, hashCode, notify, notifyAll, wait, wait, wait`

- - ### Constructor Detail


    - #### LinearNNSearchSkipInstanceItselfTest

      ```
      public LinearNNSearchSkipInstanceItselfTest()
      ```
  - ### Method Detail


    - #### testGetNeighbours

      ```
      public void testGetNeighbours()
      ```

      Test method for
      `main.ml2db.al.utility.neighbours.LinearNNSearchSkipInstanceItself#getNeighbours(weka.core.Instance, uk.ac.ed.inf.enzml.mulan.mulanarff.IdMultiLabelInstances, int, boolean)`
      .


    - #### testKNearestNeighboursInstanceIntBoolean

      ```
      public void testKNearestNeighboursInstanceIntBoolean()
      ```

      Test method for
      `LinearNNSearchSkipInstanceItself.kNearestNeighbours(weka.core.Instance, int, boolean)`
      .


    - #### testLinearNNSearchSkipInstanceItself

      ```
      public void testLinearNNSearchSkipInstanceItself()
      ```

      Test method for
      `main.ml2db.al.utility.neighbours.LinearNNSearchSkipInstanceItself#LinearNNSearchSkipInstanceItself(uk.ac.ed.inf.enzml.mulan.mulanarff.IdMultiLabelInstances)`
      .


- Overview
- Package
- Class
- Use
- Tree
- Deprecated
- Index
- Help

- Prev Class
- Next Class

- Frames
- No Frames

- All Classes

- Summary:
- Nested |
- Field |
- Constr |
- Method

- Detail:
- Field |
- Constr |
- Method
